# Development of a colloidal gold-based immunochromatographic assay for rapid detection of nasal mucosal secretory IgA against SARS-CoV-2

**DOI:** 10.3389/fmicb.2024.1386891

**Published:** 2024-05-30

**Authors:** Baoqing Sun, Zhilong Chen, Bo Feng, Si Chen, Shilin Feng, Qian Wang, Xuefeng Niu, Zhengyuan Zhang, Peiyan Zheng, Ming Lin, Jia Luo, Yingxian Pan, Suhua Guan, Nanshan Zhong, Ling Chen

**Affiliations:** ^1^State Key Laboratory of Respiratory Disease, Guangzhou Institute of Respiratory Health, The First Affiliated Hospital of Guangzhou Medical University, Guangzhou, China; ^2^Guangzhou Laboratory, Guangzhou, China; ^3^Xiamen United Institute of Respiratory Health, Xiamen, China; ^4^Xiamen Fortune Bio. Co., Ltd, Xiamen, China; ^5^Guangzhou nBiomed, Guangzhou, China; ^6^Guangzhou Institute of Biomedicine and Health, Chinese Academy of Sciences, Guangzhou, China

**Keywords:** COVID-19, mucosal immunity, omicron, reinfection, SARS-CoV-2, secretory IgA (SIgA), spike

## Abstract

**Introduction:**

Infection with SARS-CoV-2 begins in the upper respiratory tract and can trigger the production of mucosal spike-specific secretory IgA (sIgA), which provides protection against reinfection. It has been recognized that individuals with high level of nasal spike-specific IgA have a lower risk of reinfection. However, mucosal spike-specific sIgA wanes over time, and different individuals may have various level of spike-specific sIgA and descending kinetics, leading to individual differences in susceptibility to reinfection. A method for detecting spike-specific sIgA in the nasal passage would be valuable for predicting the risk of reinfection so that people at risk can have better preparedness.

**Methods:**

In this study, we describe the development of a colloidal gold-based immunochromatographic (ICT) strip for detecting SARS-CoV-2 Omicron spike-specific sIgA in nasal mucosal lining fluids (NMLFs).

**Results:**

The ICT strip was designed to detect 0.125 μg or more spike-specific sIgA in 80 μL of NMLFs collected using a nasal swab. Purified nasal sIgA samples from individuals who recently recovered from an Omicron BA.5 infection were used to demonstrate that this ICT strip can specifically detect spike-specific sIgA. The signal levels positively correlated with neutralizing activities against XBB. Subsequent analysis revealed that people with low or undetectable levels of spike-specific sIgA in the nasal passage were more susceptible to SARS-CoV-2 reinfection.

**Conclusions:**

This nasal spike-specific sIgA ICT strip provides a non-invasive, rapid, and convenient method to assess the risk of reinfection for achieving precision preparedness.

## 1 Introduction

Since the outbreak of SARS-CoV-2 at the end of 2019, the virus has evolved to produce many variants aimed at increasing transmission and evading vaccine or infection-induced immunity. Since 2021, the dominant circulating strains have been the Omicron subvariants including BA.1 in 2021, BA.5 in 2022, XBB, BA.2.86, and JN.1 in 2023, among others. Despite the popular use of a range of intramuscularly injected vaccines, many individuals have experienced multiple infections (Pulliam et al., 2022; Tan et al., 2023). A study revealed that people in the USA had experienced reinfections increased substantially from the Delta (2.7%) to the Omicron (28.8%) periods (Ma et al., 2023). Reinfection could increase the risk of death, hospitalization, and long-term health issues, particularly among elderly individuals and those with pre-existing medical conditions such as diabetes (Mantovani et al., 2020; Bowe et al., 2022). At least 65 million individuals worldwide are estimated to have long COVID, and more than 200 symptoms have been identified with impacts on multiple organs (Davis et al., 2023). Given that infection-induced immunity wane over time, it would be of great value to know the risk of reinfection ahead of time. This knowledge would enable individuals at risk to better prepare for personal protection or to take other preventive measures.

SARS-CoV-2 especially Omicron subvariants preferentially infect nasal epithelial cells via the receptor binding domain (RBD) of spike protein to interact with the angiotensin-converting enzyme-2 (ACE-2) receptor on cell surface (Walls et al., 2020). The spike protein is a key antigen for eliciting antibodies, particularly neutralizing antibodies specific against SARS-CoV-2 (Yang and Du, 2021). Intramuscularly injected COVID-19 vaccines have limited capability in preventing infection since they induce antigen specific IgG antibodies only in the blood but not sIgA on mucosal surface in upper respiratory tract (Lund and Randall, 2021). People who have “hybrid immunity,” i.e., vaccination plus a natural infection have less chance to have a reinfection. Mucosal immune responses in the respiratory tract are vital for early restriction of viral replication and the clearance (Froberg and Diavatopoulos, 2021). SARS-CoV-2 infection can induce mucosal spike-specific sIgA that prevent viral invasion and reinfection (Sterlin et al., 2021; Tang et al., 2022; Wright et al., 2022; Wang et al., 2023). Several studies have reported that spike-specific mucosal IgA in bronchoalveolar lavages, saliva, or nasal swab samples can be detected in people who recovered from a SARS-CoV-2 infection (Sterlin et al., 2021; Havervall et al., 2022; Tang et al., 2022). Mucosal sIgA is produced locally and is present as dimeric and multimeric forms that can block the virus entry via upper respiratory tract. It has been reported that individuals with high level of mucosal spike-specific sIgA in nasal passage or salivary had a lower risk of Omicron breakthrough infection, a correlation not observed with IgG (Havervall et al., 2022; Marking et al., 2023a). However, the spike-specific sIgA in nasal mucosa wanes by 9 months after infection (Liew et al., 2023). Therefore, detection of spike-specific sIgA in the nasal passage may serve as a good indicator for estimating the risk of reinfection. Here, we developed and evaluated an ICT strip for rapid detection of spike-specific sIgA in the nasal passage.

## 2 Materials and methods

### 2.1 Ethical approval

This study was approved by the Medical Ethics Committee of the First Affiliated Hospital of Guangzhou Medical University (reference number 2023039-01).

### 2.2 Cloning, expression, and purification of SARS-CoV-2 spike protein

The ectodomain of spike protein of Omicron XBB was synthesized and cloned into pSecTag2A plasmid vector. The plasmids were transfected into the Expi293 cells using PEI (PolyScience) for expression of spike protein. Spike protein in the culture media was purified using Ni sepharose (Cytiva) affinity chromatography.

### 2.3 Preparation of colloidal gold and gold conjugated monoclonal antibody

Colloidal gold was prepared by trisodium citrate method (Sun et al., 2018). Briefly, 4 mL of 1% chloroauric acid was added to the Erlenmeyer flask with 96 mL double distilled water which was stirring and heating, followed by the rapid addition of 11.5 mL of 1% trisodium citrate solution with rapid stirring. The mixture was boiled for another 5 min and gradually boiled until the color gradually changes from light yellow to deep red and no longer changes in color. The colloidal gold solution was cooled to room temperature and then stored at 4°C. 0.1 M potassium carbonate was added to adjust the pH of the colloidal gold solution to 8.5. A mouse anti-human IgA monoclonal antibody (mAb) was purchased from Guiyang Keda Ltd. The binding epitope of this mAb is the Fc region of IgA. 20 μg of this antibody was added into 1 mL colloidal gold solution and incubated for 30 min. The 10% bovine serum albumin (BSA) was added to the colloidal gold conjugation and incubated for another 30 min. The mixture was then centrifuged at 10,000 × g, 4°C for 30 min to remove any unbound antibody. The pellet was resuspended in phosphate-buffered saline (PBS) containing 1% BSA.

### 2.4 Preparation of the colloidal gold-based immunochromatographic strip

**Figure 1:**
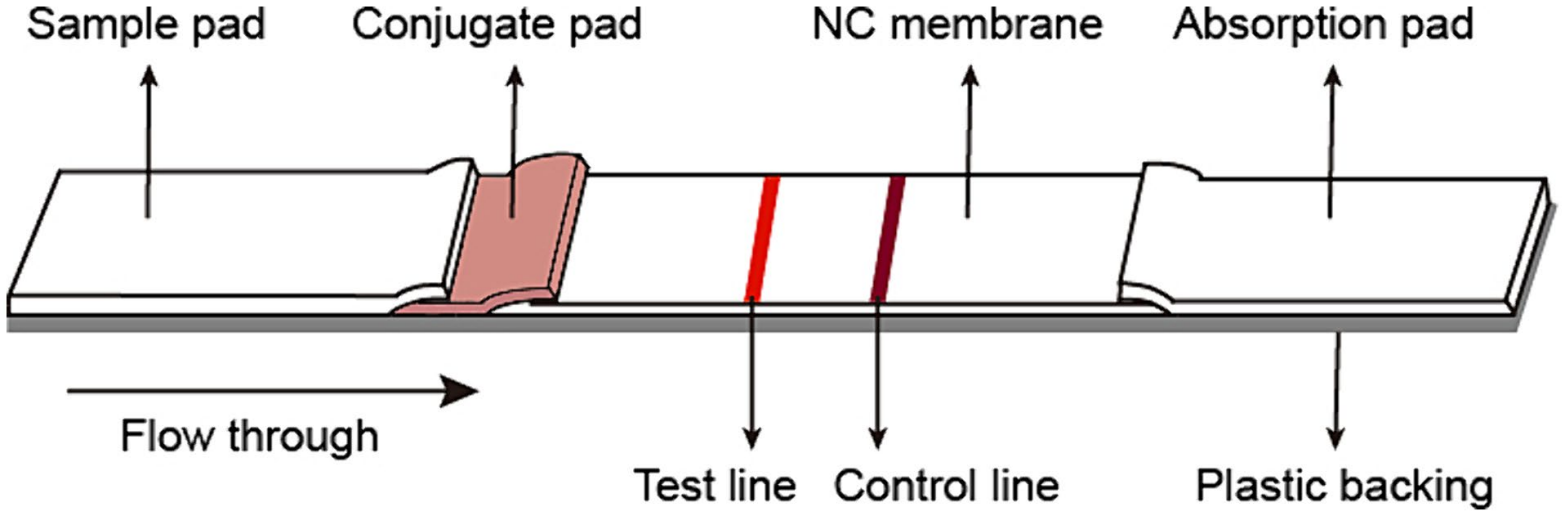
Schematic diagram of the immunochromatographic strip.

The fiberglass sample pad, conjugate pad, nitrocellulose membrane, and absorb pad were assembled on the support board sequentially, with 1–2 mm overlapping each other and cut into 3 mm pieces (Autokun) to form an immunochromatographic (ICT) strip. Briefly, the fiberglass pad was saturated with 10% BSA, and dried at 37°C overnight. The colloidal gold conjugated antibody was dispensed on the fiberglass pads to generate conjugated pads (Figure 1). The conjugated pads were dried at 37°C for 4 h. On a 2.5 cm nitrocellulose membrane, the spike protein was dispensed on the Test line (T), while the goat-anti mouse IgG was dispensed on the Control line (C). The excess mouse mAb-colloidal gold complex that passes the Test (T) line was captured by the goat anti-mouse IgG on the Control (C) line, validating the quality of the strip. The nitrocellulose membrane was dried at 37°C at least 4 h. Pure cellulose fiber was used as an absorbent pad. Immunochromatographic strips were store in a desiccator at 25°C prior to use. The ICT strip was designed to be “less sensitive,” i.e., only to detect the presence of 0.125 μg or more spike-specific sIgA in 80 μL, which is equivalent to 1.56 μg/mL or 4.1 nM spike-specific sIgA.

### 2.5 Quantification of signal value on the ICT strips

A colloidal gold immunochromatographic card reader (FIC-Q1, Autokun) was used to quantify the photoelectric signal value on the Test line (T) and Control line (C). The T value and C value were analyzed using HMReader software.

### 2.6 Evaluation of specificity and sensitivity of the sIgA detection strip

To evaluate the specificity of the ICT strip, we used purified nasal sIgA and serum IgG from Omicron BA.5 convalescents, nasal lavage fluids from Omicron BA.5 convalescents, and nasal lavage fluids from convalescents of influenza virus H1N1, H3N2, H5N6, and H7N9. An aliquot of 80 μL of each sample containing sIgA or IgG antibody was added onto the ICT strips and incubated for 15 min at room temperature. Sensitivity was determined using a serial diluted of purified nasal sIgA and mAb 714-1 sIgA, a sIgA monoclonal antibody that specifically bind to all spike proteins. The samples were two-fold serially diluted using 0.01 M Tris buffer with concentrations ranging from 0.78 μg/mL to 37.5 μg/mL. An aliquot of 80 μL of each dilution was added onto the ICT strip.

### 2.7 Collection of nasal swab samples

Nasal swab samples were collected from individuals with or without history of SARS-CoV-2 infection. All donors, 18–60 years old, received 2 doses of inactivated whole-virus vaccine more than 1 year ago. Nasal swab samples from uninfected individuals were collected before November 2022 when China was under strict zero-COVID policy. These individuals were weekly tested by PCR and were all negative for SARS-CoV-2. Nasal swab samples from infected individuals were collected between 1–2 and 4–5 months after infection with Omicron BA.5 in December 2023. Infections were confirmed by PCR or antigen test. To collect nasal mucosal lining fluids (NMLFs), a disposable nasal swab was inserted into nostrils and circulated for 30 rounds to ensure the nasal swap was saturated with NMLFs. The nasal swab was dispensed in 1 mL Tris–HCl (10 mM, pH 8.3) buffer with 150 mM NaCl and subjected to ICT strip detection. The signal values on the Test line (T) and Control line (C) were determined by a colloidal gold immunochromatography card reader (FIC-Q1, Autokun).

### 2.8 Collection of nasal lavage fluids and purification of nasal sIgA

The nasal lavage fluids were collected from SARS-CoV-2 convalescents by washing nasal passage with 200 mL saline using a nasal wash irrigator. Nasal IgA was purified via affinity chromatography using peptide M-coupled agarose beads (InvivoGen). Purified nasal sIgA was concentrated using a 100 kDa ultrafiltration tube (Merck Millipore), and the buffer was replaced with PBS. The concentration of the purified IgA was determined using a BCA protein assay kit (Thermo Fisher Scientific).

### 2.9 Enzyme-linked immunosorbent assay

SARS-CoV-2 variants spike protein or influenza HA protein were, respectively, coated onto 96-well enzyme-linked immunosorbent assay (ELISA) plate at 100 ng/well in 0.1 M carbonate buffer (pH 9.6) overnight at 4°C and blocked with blocking buffer (PBS, 0.05% Tween-20, 5% milk) at 37°C for 2 h. 4-fold dilutions of nasal swab samples in blocking buffer were incubated with antigen-coated plates for 2 h. After wash 3 times with PBST (PBS with 0.05% tween 20), HRP-conjugated secondary antibodies were incubated for 1 h, and washed with PBST. The plates were incubated with TMB (Millipore) solution in dark for 15 min. Reactions were stopped with 1 M H_2_SO_4_ and measured absorption at 450 nm. The cutoff value was defined as 2.1-fold of OD450 values from the sample of nasal swab sample diluent.

### 2.10 Pseudoviruses neutralization assay

Pseudovirus neutralization assays were performed based on a previous report (Nie et al., 2020). Briefly, 4-fold serially diluted 719–1 sIgA mAb or purified total sIgA were incubated with the SARS-CoV-2 XBB luciferase pseudovirus (500 TCID50/well) for 1 h 37°C. 100 μL of the mixtures were added to 100 μL of 293 T-hACE2 cells (2 × 10^4^/ml) in the 96-well plate and cultured at 37°C with 5% CO2 for 72 h. Cells were lysed, and the luciferase signal was measured by the Bio-Lite Luciferase Assay System (Vazyme). The data was analyzed by GraphPad Prism 8 using nonlinear regression and to calculate the 50% inhibitory concentration (IC50).

### 2.11 Statistical analysis

For statistical analyses in which two groups with normally distributed date were compared, an unpaired Student’s t test was used. All statistical analysis was performed using GraphPad Prism 8 or SPSS 12.0.

## 3 Results

### 3.1 Production and purification of the recombinant spike protein of SARS-CoV-2 omicron XBB

**Figure 2:**
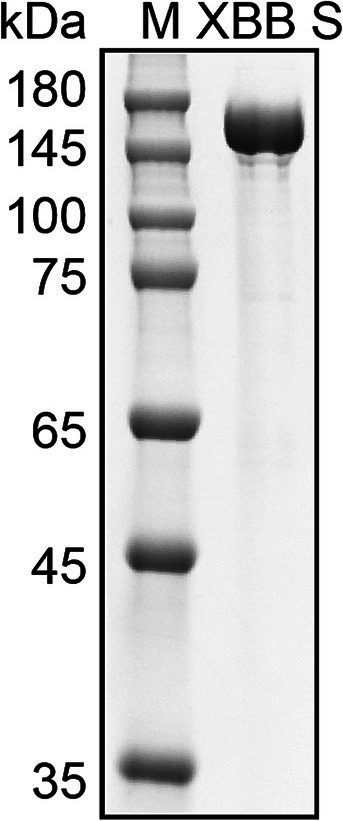
Purification of Omicron XBB Spike protein. SDS-PAGE analysis of spike protein purified from the cell cultured media of Expi293 cells transfected with His tagged XBB S plasmid. M, molecular weight marker.

We utilized the ectodomain of the Omicron XBB spike as the target antigen for detecting spike-specific sIgA, which was a major circulating strain worldwide in 2023 and has 99% amino acids identical to BA.5, 96% amino acids identical to BA.2.86, and 96% amino acids identical to latest Omicron subvariant JN.1. The recombinant ectodomain of the XBB spike was expressed in Expi293 cells and purified using affinity chromatography. The spike protein was eluted in PBS containing 300 mM imidazole. SDS-PAGE confirmed that the purity of the eluted protein was over 95% (Figure 2).

### 3.2 Development of an immunochromatographic strip for detecting spike-specific sIgA

A mouse anti-human IgA monoclonal antibody (mAb) was conjugated with colloidal gold and dispensed onto fiberglass pads. The SARS-CoV-2 spike protein was diluted to 1.2 mg/mL in phosphate buffer and coated on the nitrocellulose membrane as the capture Test line. Then the goat anti-mouse IgG antibody was diluted to 0.3 mg/mL in phosphate buffer and dispensed on the nitrocellulose membrane as control line. The spike specific sIgA can be detected by forming gold conjugated mAb-sIgA-spike sandwich complex.

**Figure 3:**
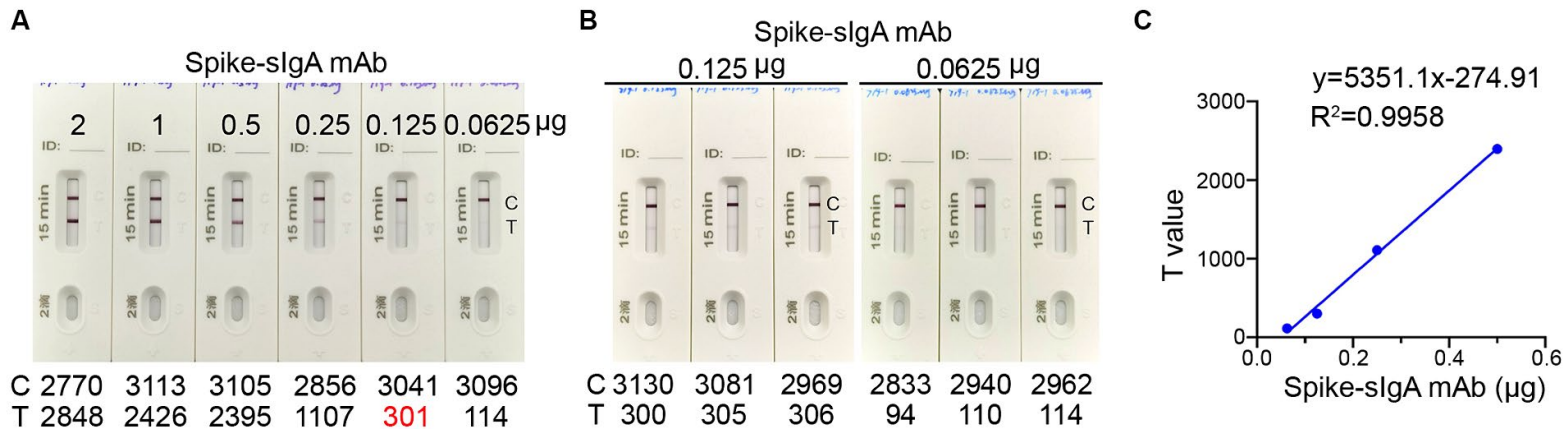
The detection limit of the ICT strip. **(A)** An aliquot of spike-specific mAb 719-1 sIgA containing 0.0625, 0.125, 0.25, 0.50, 1.00, and 2.00 μg mAb 719-1 sIgA in 80 μL solution was added to the ICT strips. These ICT strips were analyzed using a colloidal gold ICT reader. **(B)** Using a colloidal gold ICT card reader to measure the photoelectric signals on repeated triplicates of ICT strips loaded with either 0.0625 μg or 0.125 μg mAb 719-1 sIgA in 80 μL solution. **(C)** Pairwise scatter plot of the spike specific sIgA monoclonal antibody and T value. Individual data are presented.

To establish a threshold for detecting spike-specific sIgA, we utilized mAb 719-1 sIgA, which was a sIgA monoclonal antibody isolated from human nasal mucosa and possesses similar binding activities to the spikes of SARS-CoV-2 variants (paper in review). This antibody was constructed in pCMV-IgA1 plasmid, and expressed with J chain and secretory component in Expi293 cells. An aliquot of each preparation containing 0.0625, 0.125, 0.25, 0.50, 1.00, and 2.00 μg mAb 719–1 sIgA in 80 μL solution was added to the ICT strips (Figure 3A). Using mAb 719–1 sIgA as a reference, we developed the ICT strip that can visibly detect 0.125 μg or more spike-specific sIgA. The presence of 0.125 μg, but not 0.0625 μg spike-specific sIgA in 80 μL resulted in a positive band on the Test line. Using a colloidal gold ICT card reader to measure the photoelectric signals on repeated triplicates, 0.125 μg spike-specific sIgA showed a value of 301–306, while 0.0625 μg spike-specific sIgA showed a value of 94–114 (Figure 3B). This ICT strip exhibited good linearity within the range of 0.0625 to 0.5 μg spike-specific sIgA, with the signal values on the Test line ranged between 114 and 2,395 (Figure 3C).

### 3.3 Specificity of the spike-specific sIgA ICT strip

**Figure 4:**
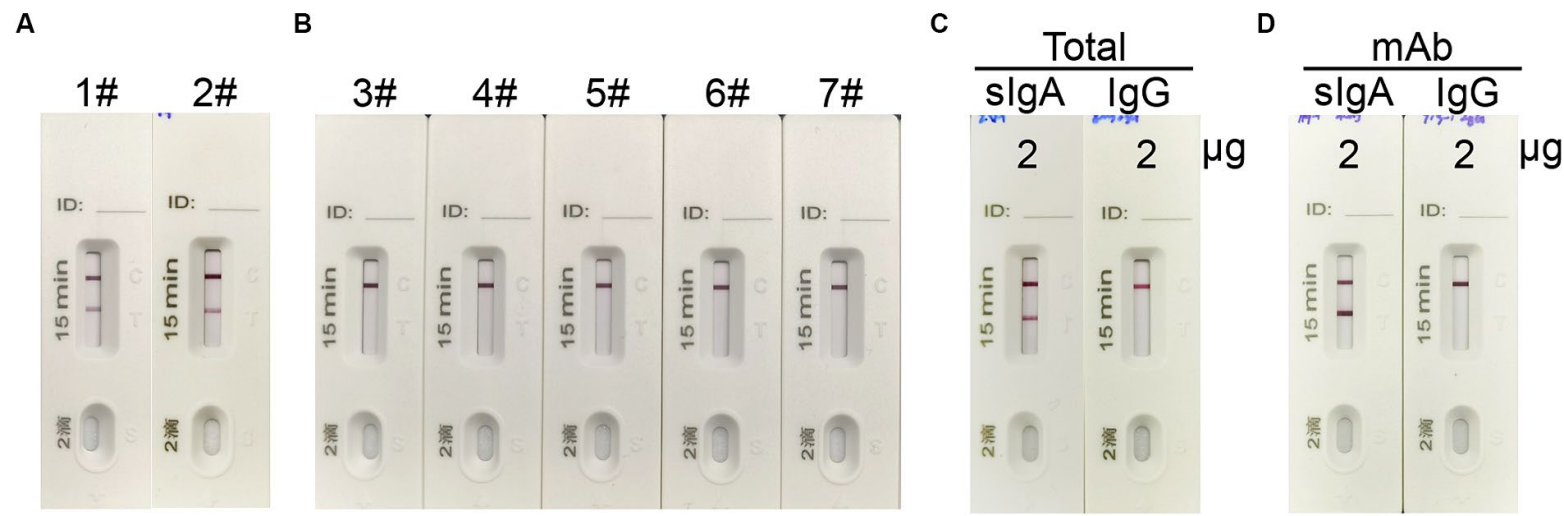
The specificity of the sIgA strip. **(A)** 1#: Nasal swab sample from an Omicron BA.5 convalescent, #2: Nasal swab sample from an Omicron XBB convalescent. **(B)** 3#–7#: Nasal swab samples from convalescents of H7N9, H1N1, H3N2, H7N9 and H5N6. **(C)** 2 μg total sIgA purified from nasal wash and 2 μg total IgG purified from serum from a SARS-CoV-2 convalescent in 80 μL were applied to the strips. **(D)** 2 μg spike specific mAb 719-1-sIgA or spike specific mAb 719-1-IgG in 80 μL were applied to the ICT strips.

To validate the specificity of the ICT strip for detecting spike-specific sIgA, we utilized nasal mucosal samples obtained from individuals who recovered from infection of Omicron BA.5 or XBB, as well as nasal mucosal samples from individuals who recovered from influenza A virus H1N1, H3N2, H7N9, and H5N6 infections. The presence of spike-specific sIgA and influenza-specific sIgA in the nasal swab samples was confirmed using an ELISA assay (Supplementary Table 1). Consistent with the ELISA results, a positive signal in the Test line was only observed in samples from SARS-CoV-2 convalescents, and not in those from influenza A virus convalescents (Figures 4A,B). To further validate if the ICT strip is specific for detecting spike-specific sIgA but not spike-specific IgG, we used purified nasal sIgA and purified serum IgG from an Omicron BA.5 convalescent. In addition, mAb 719–1 sIgA and its isoform, mAb 719–1 IgG, were also used for validation. Application of 2 μg of purified nasal sIgA or serum IgG from an Omicron convalescent, or 2 μg of mAb 719-1 sIgA or mAb 719-1 IgG in 80 μL sample buffer to the ICT strips showed that sIgA showed a positive band in the Test line, whereas IgG samples showed no positive band in the Test line (Figures 4C,D). Therefore, this ICT strip is specific for detecting spike-specific sIgA and is not interfered by spike-specific IgG.

### 3.4 Correlation of spike-specific sIgA with neutralizing activities

**Figure 5:**
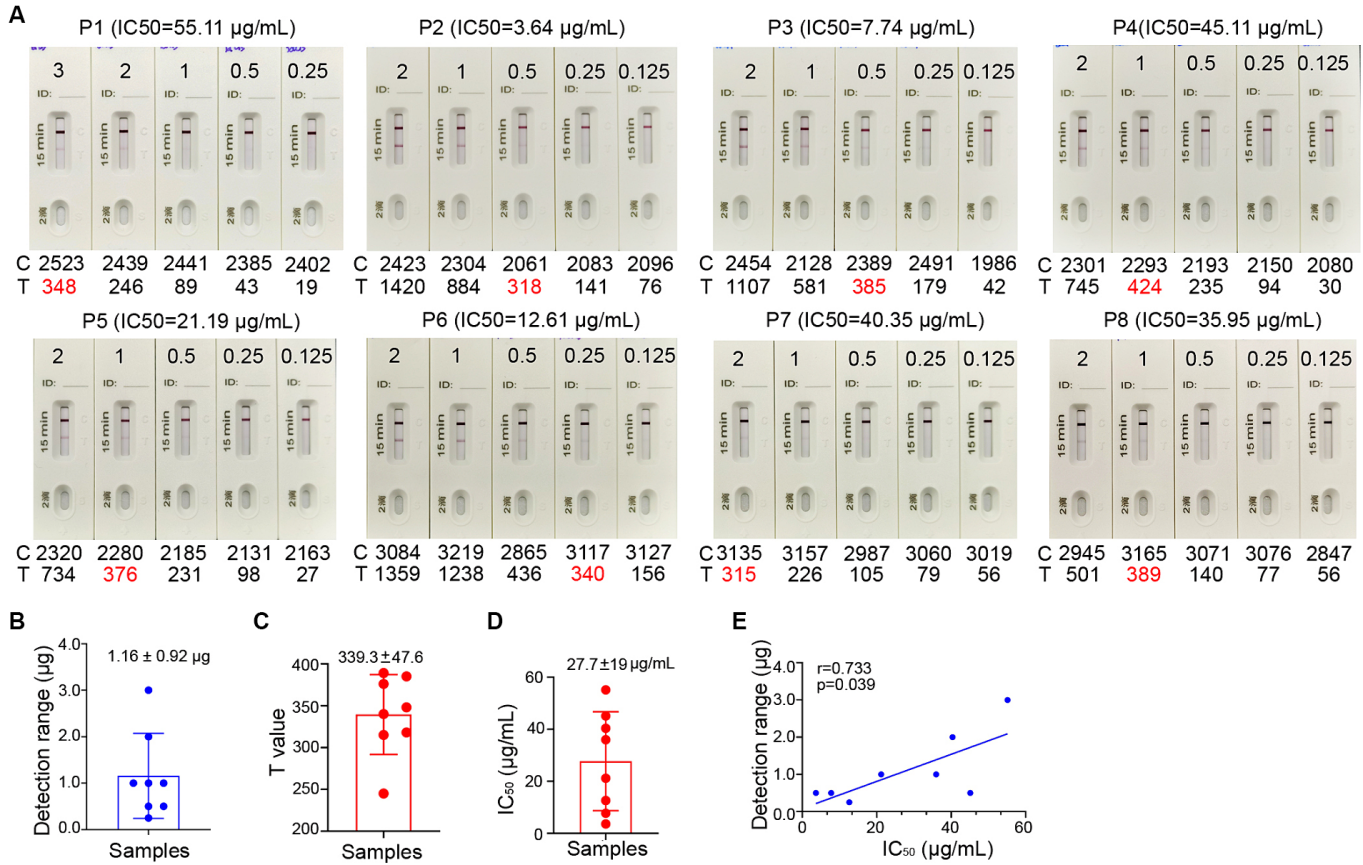
Correlation between the signal level on the Test line of the ICT strip and neutralizing activity. **(A)** Two-fold serial dilutions of purified nasal sIgA from 8 convalescents (P1-8) ranging from 1.563 μg/mL to 37.50 μg/mL. Each strip was loaded with 80 μL diluted samples that containing 0.125 to 3.00 μg total sIgA. Samples were tested on the strips and were also analyzed by colloidal gold immunochromatography analyzers. **(B)** Minimal total nasal sIgA from Omicron BA.5 convalescents that provides a visible band on the Test line of the ICT strips in panel **(A)**. **(C)** Using a colloidal gold immunochromatographic card reader to measure the photoelectric signal on the Test line of the ICT strips in panel **(A)**. **(D)** The 50% inhibitory concentrations (IC50) in neutralization against XBB for 8 convalescents (P1-8). **(E)** Pairwise scatter plot of the IC50 and the amount of total nasal sIgA for 8 convalescents (P1-8). Data are shown as mean ± SD (*n* = 8). Individual data are presented.

In general, a SARS-CoV-2 reinfection is defined, according to national surveillance guidance, as a positive outcome from a PCR or antigen test conducted on a respiratory specimen that is collected more than 90 days following a previous confirmed or probable COVID-19 case in the same individual (Pilz et al., 2022). To estimate the level of spike-specific sIgA in the nasal passage that may predict the risk of reinfection, we purified sIgA from NMLFs obtained from 8 healthy donors at 1–2 months after an Omicron BA.5 infection (Supplementary Table 2). An aliquot of each preparation containing 0.125, 0.25, 0.50, 1.00, 2.00, and 3.00 μg total sIgA in 80 μL solution was added to the ICT strips (Figure 5A). The visible signal on the Test line could be observed in the strips loaded with 1.16 ± 0.92 μg total sIgA (range 0.25–3.0 μg; Figure 5B; Supplementary Table 3). Using a colloidal gold ICT reader, the photoelectric signal values on the Test line were obtained. The range of minimal visible signal in the T line had a mean value of 339.3 ± 47.6 (Figure 5C; Supplementary Table 3). We used mAb 719-1 sIgA as a reference standard to quantitate the amount of spike-specific sIgA in these 8 samples. At least 0.11 ± 0.01 μg spike-specific sIgA need to be present in 0.25–3.0 μg total sIgA to generate a visible signal (Supplementary Table 3).

To determine whether the level of spike-specific sIgA correlates with the neutralizing activities in the samples, we assessed the neutralizing titers of purified sIgA against Omicron XBB using a pseudovirus virus neutralization assay. These samples exhibited 50% inhibitory concentrations (IC50) of 27.7 ± 19 μg/mL of total nasal sIgA for samples collected at 1–2 months after infection (Figure 5D; Supplementary Table 3). Correlation analysis showed that the level of spike-specific sIgA positively correlated with the neutralizing activities (r = 0.733, *p* = 0.039; Figure 5E), suggesting that the signal value on the ICT strip can reflect the neutralizing potency of nasal sIgA. A sample with a high intensity band on the Test line indicates great potency in neutralizing SARS-CoV-2.

### 3.5 Testing nasal swab samples in people with or without history of SARS-CoV-2 infection

We used nasal swabs to collect NMLFs and resolved each nasal swab in 1.0 mL dispensing solution. ELISA quantification showed that there were 333 ± 175 μg/mL total IgA in 1.0 mL nasal swab dispensing solution (Supplementary Table 4), which falls into the range of calculated concentration of 410 ± 116 μg/mL total sIgA in the nasal passage detected by washing the nostrils using 1,000 mL saline (paper in review). Therefore, detecting the amount of spike-specific sIgA in 1.0 mL nasal swab dispensing solution can estimate the amount of spike-specific sIgA in the nasal passage.

We randomly collected 103 nasal swab samples from convalescents at 1–2 months after a BA.5 infection. For negative control, we collected 92 nasal swab samples from people without history of SARS-CoV-2 infection. All nasal swab samples from convalescents showed a positive signal on the Test line, whereas all nasal swab samples from uninfected people tested negative on the Test line (Supplementary Table 5). We validated the sensitivity and specificity of the ICT strip using ELISA to detect spike-specific sIgA in the same samples. We collected 87 nasal swab samples from SARS-CoV-2 convalescents at 4–5 months after a BA.5 infection and 92 nasal swab samples from people without history of SARS-CoV-2 infection. Of note, the ICT strip was intentionally designed to detect the presence of 0.125 μg or more spike-specific sIgA in 80 μL. The result showed that 83 out of 87 nasal swab samples from convalescents were positive for having 0.125 μg or more spike-specific sIgA in 80 μL NMLFs. We also used ELISA which typically exhibits greater sensitivity than ICT assay. All 87 positive samples were positive for spike-specific sIgA in the ELISA. All 92 nasal swab samples from people without history of SARS-CoV-2 infection had no detectable spike-specific sIgA in ICT assay and ELISA (Supplementary Table 6). Therefore, the specificity of this ICT strip is 100% compared to conventional ELISA (Supplementary Table 6), indicating that this ICT strip is reliable in detecting the presence of 0.125 μg or more spike-specific sIgA in 80 μL NMLFs, which can be translated to 1.56 μg /mL or more spike-specific sIgA in the nasal passage.

### 3.6 Low level of spike-specific sIgA in the nasal passage is associated with high risk of omicron reinfection

**Figure 6:**
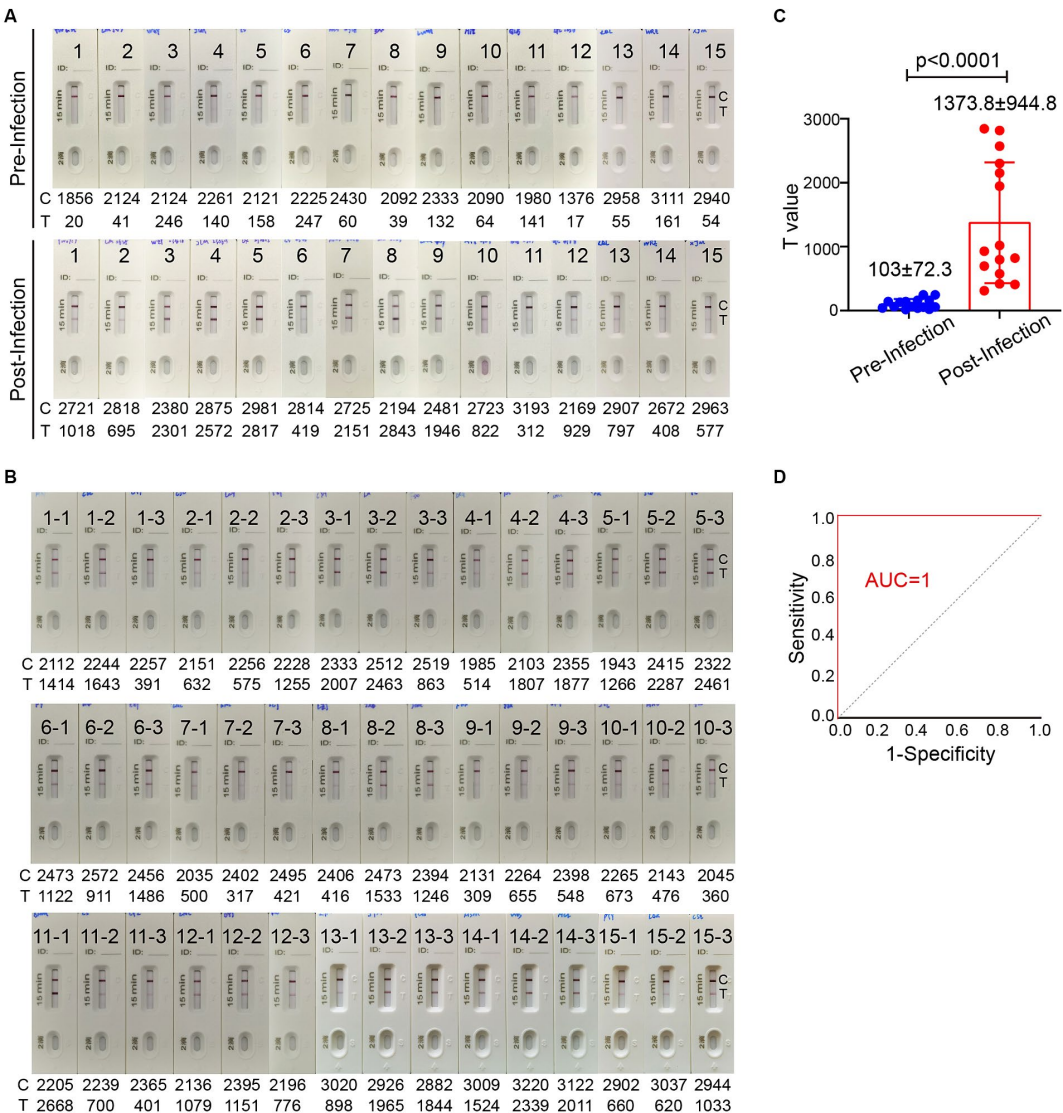
The level of spike-specific sIgA in the nasal passage and the risk of Omicron reinfection. **(A)** Nasal swab samples were collected from 15 individuals 1–2 month before Omicron reinfection (top) and 1–2 months after recovery (bottom). **(B)** Nasal swab samples from 45 people who have closed contacts with these 15 infected individuals but without infection. These samples were collected when the 15 people were having an active infection. All ICT strips were analyzed by colloidal gold immunochromatographic reader. **(C)** Statistic of T values in panel **(A)**. **(D)** The receiver operating characteristic (ROC) curve of T value in panels **(A,B)**. The area under the curve (AUC) values for the ICT strip was 1.0. Data are shown as mean ± SD, two-tailed t-test, *p* < 0.0001. Individual data are presented.

Sufficient level of spike-specific sIgA is crucial for effective protection against SARS-CoV-2 infection via upper respiratory tract especially nasal passage. It has been reported that spike-specific IgA in the nasal passage wanes to baseline in 9 months after infection (Liew et al., 2023). To investigate the association of the level of spike-specific IgA in the nasal passage with the risk of reinfection, the ICT strip was used to evaluate nasal swab samples from 140 donors who experienced an Omicron BA.5 infection after the lift of zero-COVID policy during December 2022 to January 2023. On 5–6 months after an infection, 84 individuals (60%) still had strong positive signal, while 56 individuals (40%) had very weak or even undetectable signal (Supplementary Figure 1). These individuals were monitored for the infection status in the following 2 months. Among 56 individuals who showed weak or undetectable signal of spike-specific sIgA, 17 individuals (30.4%) reported an infection during an XBB wave in May–June, 2023. In contrast, none of the 84 individuals who had relatively high level of spike-specific sIgA reported an infection during the same period. We were able to collect 15 pairs of nasal swab samples before and after the reinfection. Using the ICT reader, the signal value on the T line of these 15 individuals had a mean value of 103 ± 72.3 (range 17–247) before reinfection (Figure 6A). This result suggested that when the level of spike-specific sIgA in nasal passage is lower than 0.125 μg in 80 μL nasal swab dispensing solution, the risk of reinfection is high. Notably, the level of spike-specific sIgA became detectable in all 15 samples after the XBB infection and the signal value on the T line increased to a mean value of 1373.8 ± 944.8 (range 312–2,843; Figures 6A,C).

We also randomly collected nasal swab samples from 3 individuals who had close contact with each of these 15 infected individuals. A total of 45 samples were collected during the period when these 15 people were having an active infection. These 45 individuals did not take special personal protection and had no symptoms of COVID-19 even they shared the same office or room with infected people. All 45 nasal swab samples showed a positive band for spike-specific sIgA signal on the Test line (Figure 6B). The signal value on the Test line of these 45 nasal swab samples ranged between 309 and 2,668, with a mean value of 1147.5 ± 687.8. The ICT strip cutoff point was selected by a receiver operating characteristic (ROC) analysis to differentiate between individuals with and without reinfection. We used the T value in Figures 6A,B to make a ROC curve. The ICT has very good discriminative power for identifying individuals with reinfection, as shown by the large area (1.0) under the ROC curve (Figure 6D). The cut-off T value according to the ICT strip ROC curve was 278, and both the sensitivity and specificity were 100%. Based on the standard curve of mAb 719–1 sIgA, the cut-off value of spike-specific sIgA was 0.103 μg. Therefore, people with a level of spike-specific sIgA higher than the cut-off value of the ICT strip in the nasal passage are likely to have a lower risk of reinfection.

## 4 Discussion

As the majority of the global population has been infected with SARS-CoV-2, the risk of reinfection leading to increased risks of death, hospitalization, and various complications, including pulmonary, cardiovascular, hematological, and diabetes-related issues, underscores the need for a convenient method to assess the risk of reinfection. In response to this need, we have developed an immunochromatographic test strip for detecting SARS-CoV-2 spike-specific sIgA in the nasal passage. The strip is designed to detect more than 0.125 μg of spike-specific sIgA in 80 μL of NMLFs, which is equivalent to 1.56 μg of spike-specific sIgA per ml of NMLFs.

Antibody assays are valuable tools for determining population seroprevalence, diagnosing past infections, studying antibody responses elicited by virus infections, evaluating vaccine immunogenicity, and establishing immune correlates of protection (Ye et al., 2021). The outcomes of these assays can help assess population vulnerability and identify individuals at risk of reinfection. SARS-CoV-2 infection stimulates production of IgM, IgG, and IgA antibodies in circulation and sIgA in the upper respiratory tract (Isho et al., 2020; Long et al., 2020; Piccoli et al., 2020; Seow et al., 2020). Most earlier studies have been focused on serum antibodies but neglect nasal mucosal antibody response. There are two primary types of assays for studying serum antibody response: (1) ELISA to measure antigen-specific binding antibodies, including IgG, IgM, and IgA; (2) virus neutralization assays based on pseudovirus or authentic virus (Sun et al., 2020; Ye et al., 2021). Although serological antibody detection is commonly used, blood collection is not favorable by most people. In addition, most of the COVID-19 vaccines are administered intramuscularly, serum antibody detection may be difficult to distinguish between responses to viral infection and vaccination. It has been reported the serum IgG antibodies are not correlated with preventing breakthrough Omicron infections (Havervall et al., 2022). While systemic antibodies contribute to decrease disease severity, mucosal sIgA in the respiratory tract, play a more prominent role in preventing SARS-CoV-2 infection and transmission (Russell et al., 2020; Focosi et al., 2022). An increasing number of studies have found that high levels of nasal spike-sIgA antibodies, whether from previous infection with the wild-type SARS-CoV-2 or the Omicron variant, can reduce the risk of breakthrough Omicron infections (Havervall et al., 2022; Marking et al., 2023a,b). Currently, the detection of nasal spike-specific sIgA antibodies is mainly done through ELISA or electrochemiluminescence methods (Wang et al., 2023). However, these methods are complex, time-consuming, expensive, and difficult to apply for individuals. Our study demonstrates that a colloidal gold immunochromatographic strip enables rapid and convenient detection of nasal IgA antibodies, facilitating the assessment of infection induced antibody responses, population vulnerability, and reinfection risk.

sIgA in the nasal passage is the first line of defense against respiratory pathogens. It has been reported that nasal sIgA provides protection against various respiratory pathogens, including SARS-CoV-2, influenza, and *Streptococcus pneumoniae* (Joseph, 2022). In mice and humans, nasal sIgA specific to influenza have been found to have a higher correlation with protection compared to serum antibodies (Chen and Quinnan, 1988; Murphy and Clements, 1989). Therefore, methods for detecting nasal sIgA can not only be used for SARS-CoV-2 spike-specific sIgA in the nasal passage, but also for the detection of other pathogen-specific sIgA antibodies, such as influenza virus. Our research has important implications for the development of diagnostic assays indicating the risk of respiratory pathogen infections. If the nasal spike-specific sIgA reduce to a low or undetectable level, the risk of reinfection is high. Therefore, this ICT strip can help people to assess the risk of reinfection and remind them to take protective measures if the risk of reinfection increases.

## 5 Conclusion

This nasal spike-specific sIgA ICT strip can be used to assess infection-induced and intranasal vaccination-induced mucosal IgA in the nasal passage, determine population vulnerability, and evaluate individuals at risk of reinfection. This ICT strip provides a rapid, non-invasive, and convenient method to assess the risk of reinfection for people indeed to achieve more precision preparedness.

## Data availability statement

The original contributions presented in the study are included in the article/Supplementary material, further inquiries can be directed to the corresponding authors.

## Ethics statement

The studies involving humans were approved by the Medical Ethics Committee of the First Affiliated Hospital of Guangzhou Medical University (reference number 2023039-01). The studies were conducted in accordance with the local legislation and institutional requirements. The participants provided their written informed consent to participate in this study.

## Author contributions

BS: Data curation, Formal analysis, Resources, Writing – review & editing, Investigation, Project administration, Supervision. ZC: Conceptualization, Data curation, Formal analysis, Investigation, Methodology, Project administration, Supervision, Validation, Visualization, Writing – original draft, Writing – review & editing. BF: Conceptualization, Data curation, Formal analysis, Investigation, Methodology, Project administration, Software, Supervision, Validation, Visualization, Writing – review & editing. SC: Data curation, Formal analysis, Investigation, Methodology, Validation, Visualization, Writing – review & editing. SF: Data curation, Formal analysis, Methodology, Validation, Writing – review & editing, Visualization. QW: Formal analysis, Writing – review & editing, Data curation, Methodology, Validation. XN: Formal analysis, Resources, Writing – review & editing, Investigation. ZZ: Validation, Writing – review & editing, Methodology. PZ: Writing – review & editing, Formal analysis, Resources. ML: Writing – review & editing, Validation. JL: Writing – review & editing, Resources. YP: Data curation, Resources, Writing – review & editing. SG: Formal analysis, Investigation, Resources, Writing – review & editing. NZ: Conceptualization, Project administration, Supervision, Writing – review & editing. LC: Conceptualization, Data curation, Formal analysis, Funding acquisition, Investigation, Project administration, Supervision, Writing – original draft, Writing – review & editing.
