# Supplementary material for: Development of a colloidal gold-based immunochromatographic assay for rapid detection of nasal mucosal secretory IgA against SARS-CoV-2
Source: Front Microbiol. 2024 May 30;15:1386891. doi: 10.3389/fmicb.2024.1386891 (PMC11177785; doi:10.3389/fmicb.2024.1386891)
Supplement: Supplementary file 1 [file Data_Sheet_1.pdf]

Supplementary Table 1. ELISA assay for hemagglutinin or spike specific IgA of nasal swab samples. The sample was diluted 4 times before testing.

ID	Convalescent patient	Antigen (ELISA OD450)				
		XBB spike	H1N1 HA	H3N2 HA	H5N6 HA	H7N9 HA
1#	Omicron BA.5	0.857	0.132	0.143	0.09	0.12
2#	Omicron XBB	1.036	0.156	0.161	0.139	0.101
3#	Influenza H7N9	0.158	0.159	0.12	0.271	0.346
4#	Influenza H1N1	0.13	0.435	0.249	0.284	0.276
5#	Influenza H3N2	0.148	0.222	0.35	0.125	0.102
6#	Influenza H7N9	0.117	0.157	0.113	0.163	0.417
7#	Influenza H5N6	0.126	0.274	0.246	0.489	0.109

Supplementary Table 2. Demographics of donors who provided nasal sIgA samples.

ID	Age (year)	Gender (F/M)	Doses of inactivated vaccine	Days after being infected with BA.5
P1	41	M	2	50
P2	27	F	2	30
P3	25	F	2	30
P4	26	M	2	35
P5	32	M	2	40
P6	60	M	2	50
P7	26	F	2	35
P8	24	F	2	40

Supplementary Table 3. The minimal amount of nasal sIgA for visible detection on the ICT strip is associated with neutralizing activity.

Sample ID	XBB Pseudovirus Neutralization IC50 (μg/mL)	Total sIgA (μg) for visible signal	XBB Spike- specific sIgA in total sIgA (μg) for visible signal	Signal on Test line
P1	55.11	3	0.116	346
P2	3.64	0.5	0.111	318
P3	7.74	0.5	0.123	385
P4	45.11	1	0.097	424
P5	21.19	1	0.122	376
P6	12.61	0.25	0.115	340
P7	40.35	2	0.110	315
P8	35.95	1	0.124	389
Mean ± SD	27.7 ± 19	1.16 ± 0.92	0.11 ± 0.01	339.3 ± 47.6

Supplementary Table 4. Concentration of total sIgA in 1.0 mL dispensing solution.

ID	Total sIgA concentration (μg/mL)
1#	111
2#	228
3#	351
4#	379
5#	632
6#	144
7#	367
8#	400
9#	566
10#	114
11#	99

12#	393
13#	594
14#	354
15#	256
Mean ± SD	333 ± 175

Supplementary Table 5. Results from nasal swab samples using ICT assay.

		ICT	
	**Total sample**	**Positive**	**Negative**
**SARS-CoV-2 convalescents**	103	103	0
**Uninfected individuals**	92	0	92
**Sensitivity**		103/103×100=100%	
**Specificity**		92/92×100=100%	

Supplementary Table 6. Comparison of sensitivity and specificity between developed colloidal gold-based immunochromatographic (ICT) assay and ELISA.

		**ICT assay**		**ELISA**	
	**Total sample**	**Positive**	**Negative**	**Positive**	**Negative**
**SARS-CoV-2 convalescents**	87	83	4	87	0
**Uninfected individuals**	92	0	92	0	92
**ICT vs ELISA Sensitivity**			83/87×100=95.4%		
**ICT vs ELISA Specificity**			92/92×100=100%		
