# Supplementary figures and images for: Development of a colloidal gold-based immunochromatographic assay for rapid detection of nasal mucosal secretory IgA against SARS-CoV-2
Source: Front Microbiol. 2024 May 30;15:1386891. doi: 10.3389/fmicb.2024.1386891 (PMC11177785; doi:10.3389/fmicb.2024.1386891)

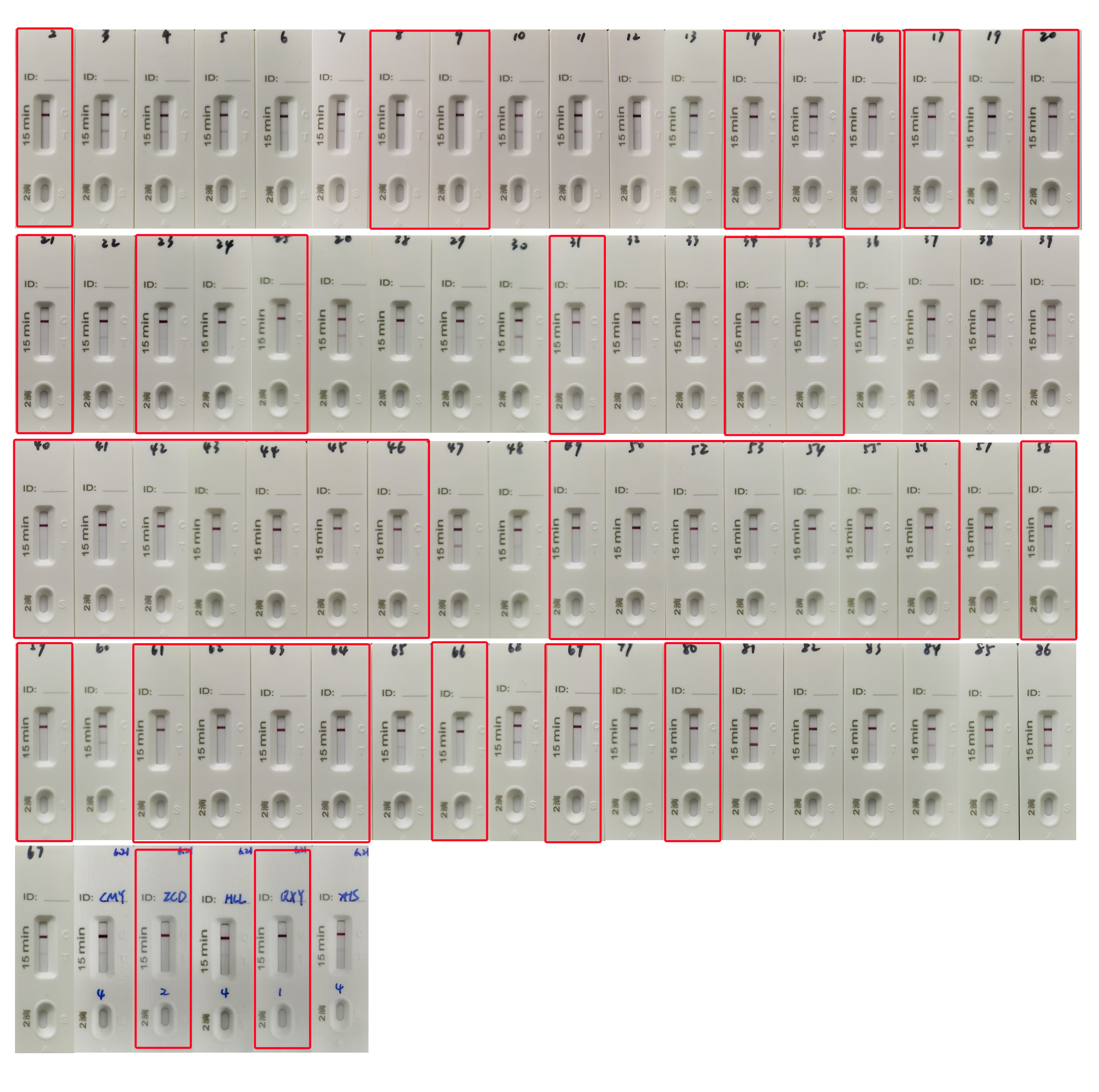

Supplement: Supplementary file 2 [file Image_1.JPEG]
